# A Biomarker Characterizing Neurodevelopment with applications in Autism

**DOI:** 10.1038/s41598-017-18902-w

**Published:** 2018-01-12

**Authors:** Di Wu, Jorge V. José, John I. Nurnberger, Elizabeth B. Torres

**Affiliations:** 1 Physics Department, Indiana University, Bloomington, Indiana, United States; 2 Stark Neuroscience Institute, Indiana University School of Medicine, Indianapolis, United States; 3 Department of Cellular and Integrative Physiology, Indiana University School of Medicine, Indianapolis, United States; 4 Key Laboratory of Theoretical Physics, Institute of Theoretical Physics, Chinese Academy of Sciences, Beijing, China; 5 Institute of Psychiatric Research, Department of Psychiatry, Indiana University School of Medicine, Indianapolis, United States; 6 Psychology Department, Rutgers University, New Brunswick, New Jersey United States; 7 Rutgers Center for Cognitive Science, Rutgers University, New Brunswick, New Jersey United States; 8 Center for Biomedical Imaging and Modeling, Computer Science Department, Rutgers University, New Brunswick, New Jersey United States

## Abstract

Despite great advances in neuroscience and genetic studies, our understanding of neurodevelopmental disorders is still quite limited. An important reason is not having objective psychiatric clinical tests. Here we propose a quantitative neurodevelopment assessment by studying natural movement outputs. Movement is central to behaviors: It involves complex coordination, temporal alterations, and precise dynamic controls. We carefully analyzed the continuous movement output data, collected with high definition electromagnetic sensors at millisecond time scales. We unraveled new metrics containing striking physiological information that was unseen neither by using traditional motion assessments nor by naked eye observations. Our putative biomarker leads to precise individualized classifications. It illustrates clear differences between Autism Spectrum Disorder (ASD) subjects from mature typical developing (TD) individuals. It provides an ASD complementary quantitative classification, which closely agrees with the clinicaly assessed functioning levels in the spectrum. It also illustrates TD potential age-related neurodevelopmental trajectories. Applying our movement biomarker to the parents of the ASD individuals studied in the cohort also shows a novel potential familial signature ASD tie. This paper proposes a putative behavioral biomarker to characterize the level of neurodevelopment with high predicting power, as illustrated in ASD subjects as an example.

## Introduction

Neurodevelopment involves the structural growth and functional maturation of the central nervous system. Humans develop a variety of brain functions through this process such as learning ability, memory, and psychomotor skills. Abnormalities during this process, either due to genetic or environmental factors, can lead to a series of neurodevelopmental disorders (NDD), including autism spectrum disorder (ASD), intellectual disability (ID), etc. In the past few years, significant progress has been made in genetics and basic neuroscience, having the promise of improving our understanding and treatments of mental illness, including NND. However, the translation has been strikingly slow: very few effective treatments or reliable clinical tests are available. A crucial challenge facing the field is the lack of scientific precision and biological relevance in current psychiatric diagnostic/classification systems^1,2^. A large proportion of the diagnostic criteria is based on the symptoms directly observable by clinicians or parents, with few biological foundations. For example, eye contact level in a social situation used in autism assessments, are dependent on cultural beliefs and social norms without a universal standard. Current symptoms-based criteria are still not reliable enough to provide insights into underlying neurobiological mechanisms to further guide genetic and neuroscience studies. There is an important need of having more objective biomedical tests to precisely monitor human neurodevelopment and disease progression, thus aiding NDD diagnosis as well as guiding further research in this area.

The traditional approach of trying to find tests with a one-to-one match to the current symptom-based psychiatric definitions is unlikely to work^3^. Instead, there is a need to focus on developing objective biological measurements to complement the current observational approach, to lay down the foundations of a more accurate psychiatric biological classifications. As part of that effort, the National Institute of Mental Health launched the “*Research Domain Criteria project”* (RDoC) to encourage the development of *precision medicine psychiatric diagnostics*^4,5^. Moreover, the nascent field of Computational Psychiatry paradigm also attempts to meet the needs for precision diagnostics by developing powerful computational and mathematical techniques^6–10^. Recent studies have made progress in objectively distinguishing patients from controls by applying machine learning techniques to fMRI neuroimaging data^11–13^. However, these approaches have certain limitations^9^: First, the features selected in these studies are in general complex and counterintuitive, with no guarantee to helping identify the underlying neural substrates. Second, the classifications in these studies do not emerge blindly from the data sets but are guided by the symptoms-based psychiatric qualitative diagnostics. Third, the classification outputs are binary, without assessing disease severity. There is still the need of identifying biologically/theoretically meaningful parameters (biomarkers) having the power to automatically characterize each individual at different neurodevelopmental stages.

Most recent research has mainly focused on studying and understanding brain circuits potentially connected to neurodevelopment and NDD. It is, however, of equal importance to study the corresponding behavioral outputs as well^14^. Understanding the behavioral outputs may provide some guidance for direct brain studies. The information contained in the behavioral outputs, especially in movements, seems to have been mostly overlooked. Some seemingly simple movements are actually produced via rather complex integrations in the sensorimotor system^15^. They arise through a complex learning processes directly related at each stage in neurodevelopment. We surmised that a detailed examination of such motion outputs may uncover some important biological information that may be related to neurodevelopment or deficits in the central nervous system.

One basic motion that a typical adult performs several times a day is reaching to a position or to an object. Goal directed reaching is learned since infancy, stabilizing through normal motor development that appears to occur between 5–7 years old, and also between 7–10 years old^16–19^. It remains a mystery how the mature sensorimotor system produces smooth motions. This despite the inevitable presence in the nervous system of different sources of stochastic noise at different temporal and spatial length scales for every motor process involved^20^. The nervous system seems to have developed strategies to compensate for the different sources of all internal noise present. Previous ASD reaching motions studies found increased trial-to-trial spatial variability^21^ and increased speed magnitude variability^22^ compared to TD individuals, suggesting increased level of noise in the ASD sensorimotor systems^23^. Here we go beyond the traditional kinematic analyses by developing novel and robust quantitative measures of the noise present in the motor outputs.

To extract such “noise”, instead of studying the trial-to-trial variability, we examined the continuous speed profiles within each motion cycle continuously at millisecond timescales. One characteristic of human movements is to have smooth bell-shaped speed profiles^24,25^. It is, however, questionable whether the visually smooth movement outputs, especially the speed profiles, are perfectly smooth with no fluctuations at all at shorter time scales, away from naked eye observations. To capture such possible fluctuations, we carefully applied a triangular smoothing algorithm to the 3D positional raw data collected with high sensitivity electromagnetic sensors. This smoothing algorithm successfully filtered out the spurious electronic noise while preserving the detailed movement physiological information in the output, in particular in the local speed fluctuations. We then developed a novel statistical analysis, specifically for this problem, of the extracted speed fluctuations. The analysis provided a new data-driven biometric, that we further validated by using numerical model simulations. The results from applying this biometric to our cohort not only support the hypothesis about the significant presence of sensorimotor noise in ASD, but it also unraveled a putative neurodevelopment biomarker. The later gives a clear quantitative distinction between ASD and mature typical developing (TD) subjects, as well as between immature and mature TD individuals. It also provides a remarkable severity quantification in the Autism Spectrum, which closely agrees with different psychiatric functioning levels clinical measures (e.g. IQ^26–28^, Vineland^29^). We also found certain correlation to modalities measuring autistic symptoms severities (ADI-R^30^, ADOS^31^). Further application of our biomarker to the ASD subjects’ parents surprisingly shows a potential filial link. We conjecture that future applications of our biometrics in larger cohorts may lead to a data-driven maladies classification providing a new diagnostic sensory-motor axis in the nascent field of Precision Psychiatry. This component has not been previously included in the DSM 5^1^ or in the Research Domain Criteria (RDoC) matrix^32^.

## Results

### Speed Profiles Differ across Subjects

**Figure 1:**
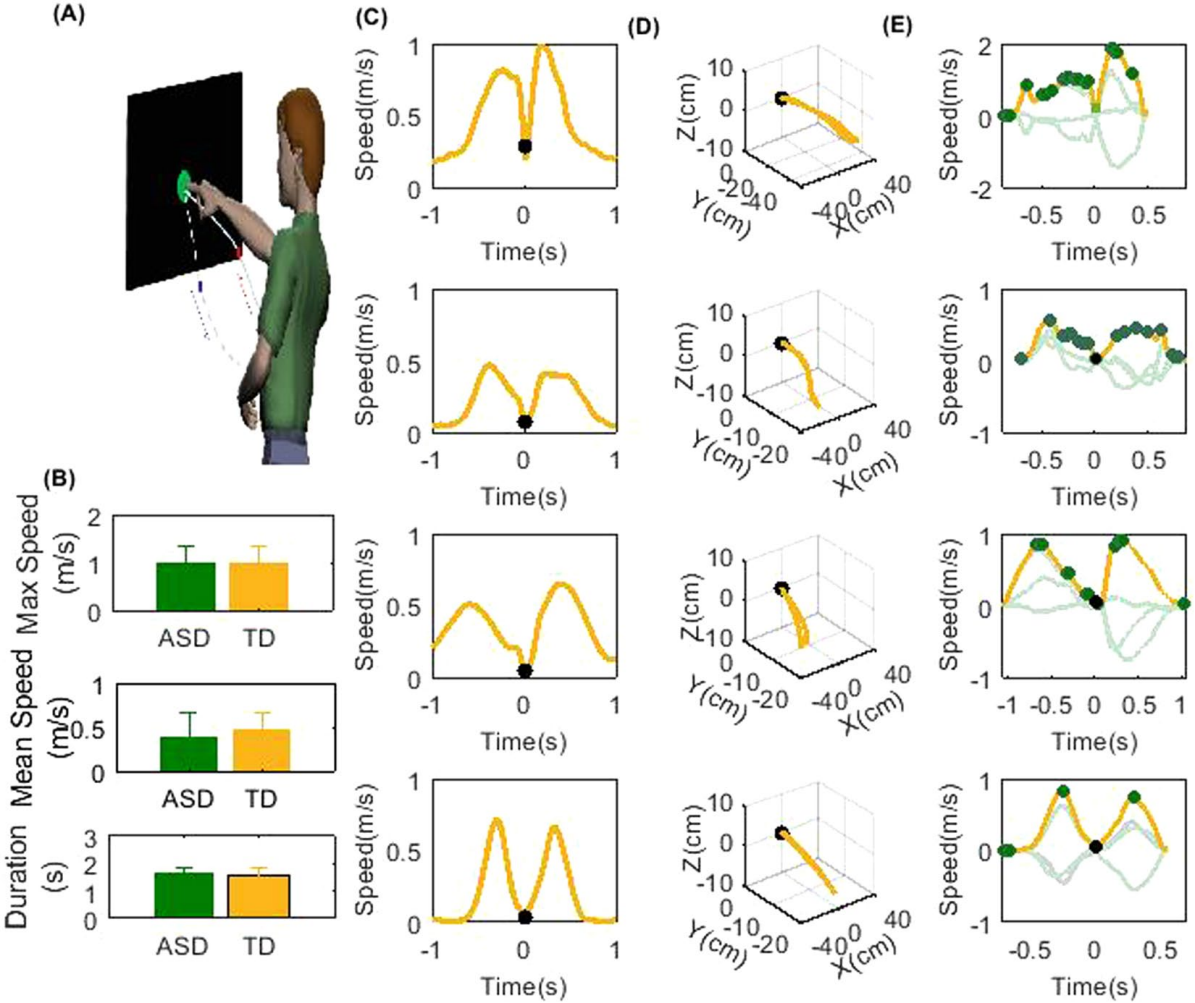
Representative hand trajectories and speed profiles during pointing task paradigm. (**A**) Schematic of the pointing task. Subjects sat comfortably in front of a touch screen, reaching out their right hands to touch the target (size 5 cm) then retracting their hands spontaneously to their resting positions. Subjects repeated the cycles continuously at their own comfortable pace (human diagram produced by EB Torres using old drawing windows software no longer available. First published in a joint paper (EBT and JVJ ref.^22^). (**B**) Compares the maximum speed magnitude, the average of the speed magnitude and the average cycle temporal durations during ASD and TD group motion cycles: there is no significant distinction among the two groups. (**C**) shows average speed profiles across cycles, lined up according to the touching points (black dots), including speed profiles in-between touching points at ±1.2 s. Results shown of three representative ASD subjects with decreasing severity (classification given in Table 1) from row 1 to row 3. Row 4 is for a TD adult. (**D**) Hand positional trajectories during one cycle for each subject defined in (**C**). (**E**) Example of velocity profiles along the three orthogonal directions (grey) with their corresponding speed profiles (yellow) during a motion cycle. Highlighted points include the target touch (black dot) and the local speed maxima (s-Peaks, green dots). Notice that the local speed fluctuations, due to the desynchronization along the three velocity directions, do not have a smooth bell shape . Please watch the subjects’ hand motion avatar movie in the Supplementary Material.

In the experiments, participants were asked to perform a basic pointing task paradigm with their dominant right hand: they reached to touch a target on a screen right in front of them, back and forth repetitively at their comfortable paces. As shown in the histograms in Fig. 1B, the average/maximum hand speed during the motion cycles, as well as the temporal length of the average motion cycle durations are not separable between ASD and TD. We used four representative subjects to illustrate our results: three ASD examples with varying levels of severities (their psychiatric clinical scores, IQ, ADI-R, Vineland Adaptive Behavior Composite, and ADOS scores, are given in Table 1) as well as one TD adult. The average speed profiles across cycles show similar bell-shape-like curves for all subjects (Fig. 1C). However, when we examined the continuous speed profiles, especially their fluctuations, within each motion cycle, at millisecond time scales, they significantly differed across subjects with ASD vs. TD adults (Fig. 1E), despite the visual indistinguishability in their corresponding positional trajectories (Fig. 1D). We named the extracted local speed peaks/fluctuations speed-Peaks or s-Peaks. These fluctuations were not visible to the unaided eyes during the experiments (as illustrated in a Supplementary Movie).

**Table 1:** Information about three representative ASD subjects with their psychiatric test scores.

	IQ	Vineland	ADI-R (sum)	ADOS	Age
ASD Case 1	N/A	19	N/A	20 (module 1)	22
ASD Case 2	71	34	45	12(module 2)	15
ASD Case 3	99	107	43	10(module 4)	25

### Triangular Smoothing Algorithm Preserves Hidden Physiological Speed Fluctuations

We applied a triangular smoothing algorithm, along each one of the three orthogonal directions, on the data collected with the electromagnetic sensors (binning window size 25 frames/104ms). The algorithm separated the external electromagnetic noise from the physiological signal (see details in Methods). We provide detailed validation for the use of our smoothing algorithm, as well as for the choice of smoothing parameters in the Supplementary Material. We also tested the robustness of our results against variations of the smoothing parameter variations and variations in external electric noise.

### Visualization of the s-Peaks

**Figure 2:**
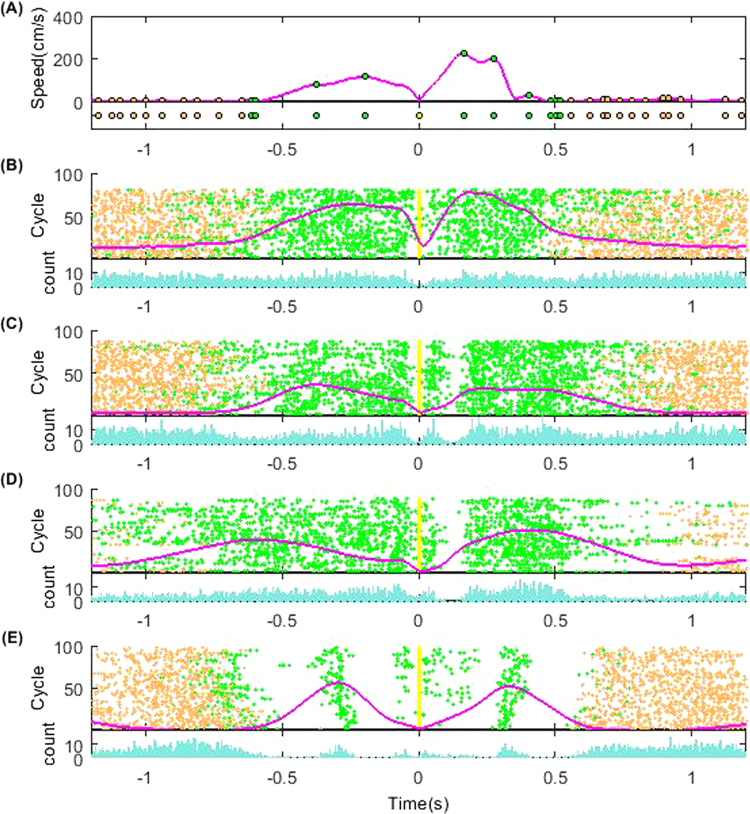
s-Peaks raster-gram-like visualizations showing their temporal micro-dynamics within a single forward-and-back motion cycle (s-Peak vector) and across motion cycles (s-Peak matrix) (**A**) s-Peak cycle vector as a function of time. Upper panel shows a single cycle hand representative speed profile from the ASD case 1 subject. The profile was centered at the touch point 0. The duration to visualize was set at touch point $\pm 1.2s$. Green dots denote local speed maxima (s-Peaks) during the motion cycle. Orange dots mark the s-Peaks outside the motion cycles (during resting periods). The bottom panel shows the s-Peak cycle vector built with the s-Peaks’ temporal information: a dot represents the appearance of an s-Peak at that time frame. (**B**–**E**) shows the s-Peak raster-gram-like matrix plotted for the four different representative cases. Each row plots the corresponding s-Peak vector per cycle, as in (**A**). The vertical axis gives the index of the trial motion cycles. The yellow dots denote the touch points during motion cycles. The magenta curve gives the averaged speed profile across trials. The bottom panel shows the s-Peak histogram counts inside each frame sampling (1/240 s).

We introduced a measure to visualize the s-Peaks’ properties and patterns in different subjects inspired by the analysis done for neuronal action potential spikes via raster-grams. We constructed s-Peaks vectors with the temporal information for each forward-and-backward cycle (Fig. 2(A)). We built s-Peaks matrices, representing the s-Peaks’ temporal patterns across cycles (Fig. 2(B–E)). We visually identified significant differences in the s-Peaks’ patterns for subjects with different psychiatric conditions. As shown in the figure, the high severity ASD representative individual (Fig. 2B) has the s-Peaks densely and randomly distributed during the multi trial sessions. In contrast, the s-Peaks in the TD adult case (Fig. 2D) shows a less chaotic structure having much fewer sporadic s-Peaks when the hand is in motion. There is a systematic increase of the s-Peaks’ randomness (decrease of the s-Peaks matrix structure) as the ASD severity level increases (Fig. 2(B,C)).

### Statistical analyses of the s-Peaks

**Figure 3:**
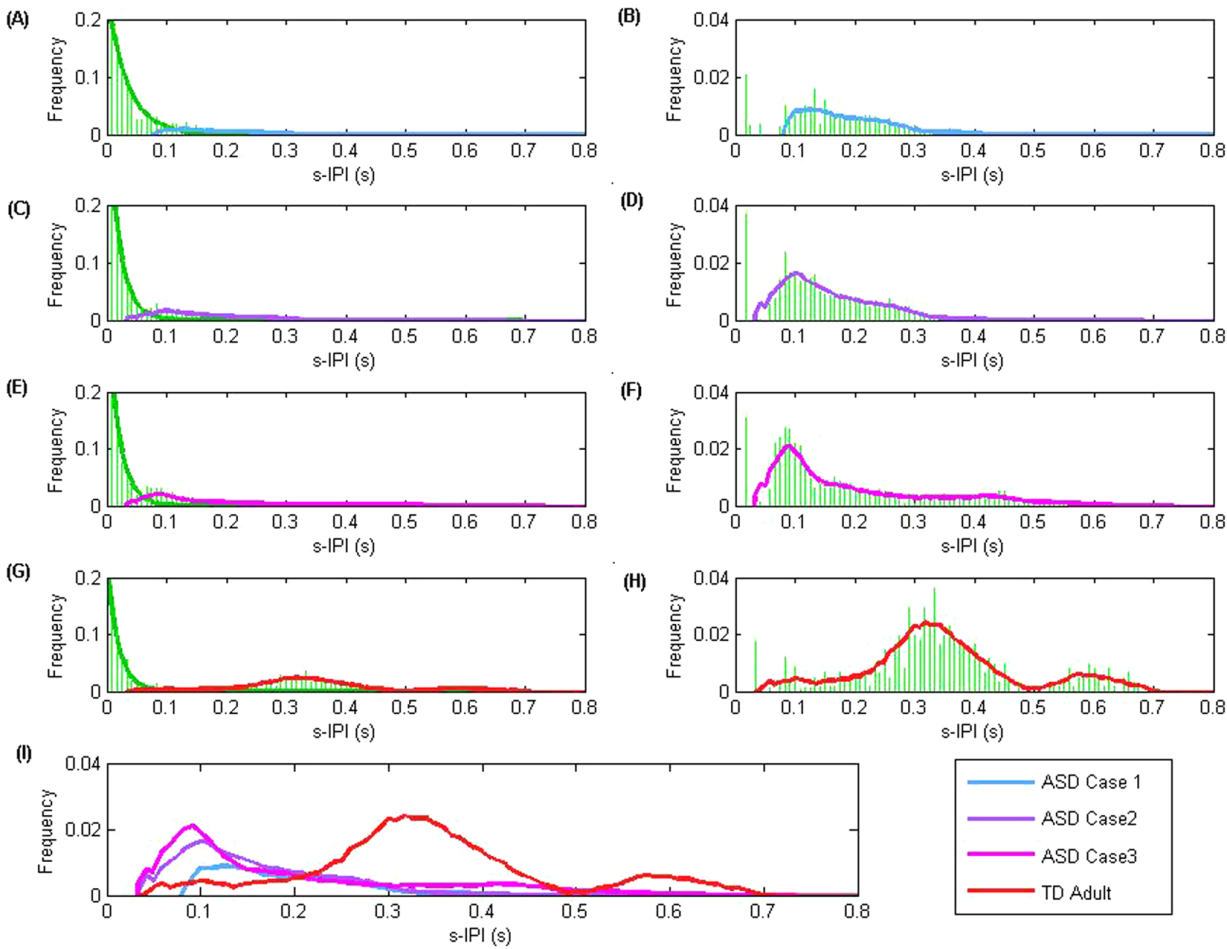
s-IPIs frequency histogram examples (**A**,**C**,**E**,**G**) shows s-IPIs frequency histograms for four representative subjects: rows 1–3 being the ASD cases; row 4 the TD adult. The histogram bin size was set at 2 frames/8 ms optimized to produce good exponential fits. Exponential fits (green lines) were applied based on the s-IPI values below 10 frames/40ms. Distribution of the residual s-IPIs outside of the exponential fit was marked with the colored lines (smoothed for visualization). (**B**,**D**,**F**,**H**) The frequency histograms of the residual s-IPIs outside the exponential fits. (**I**) Comparison of the residual s-IPI distributions across the four representative subjects: notice the systematic changes in the ASD cases and their clear distinction from the TD case.

In analogy with computational neuroscience we can quantitatively characterize the randomness of the s-Peaks by considering the statistical properties of the nearest neighbor temporal intervals between s-Peaks (speed-inter-peak-intervals or s-IPIs). Exponentially distributed s-IPIs denote total randomness in the corresponding s-Peak’s distributions. The non-exponential contributions to the s-IPIs distributions characterize s-Peaks’ “deviations from full randomness”. Figure 3 plots the calculated s-IPIs distributions as well as their non-exponential contributions for the representative cases. As shown in Fig. 3I, the non-exponential contributions in the s-IPIs distributions represent the systematic differences across different subjects. The highest severity case (ASD case 1) has almost all the s-IPIs exponentially distributed, indicating s-Peaks total randomness. The non-exponential contributions increase as the ASD severity decreases, and more so in the TD adult case.

### R-parameter Quantifies the Randomness of s-Peaks

We define here a new “R-parameter” to quantify the s-IPI deviations from full randomness. R measures the non-exponential contributions to the s-IPIs distributions. If R = 0, all the s-IPIs are distributed exponentially, suggesting total randomness in the s-Peaks’ appearances. R increases as the non-exponential contributions increase. The unbiased non-exponential contribution to s-IPI cannot distinguish, for example, one case with two small outlier intervals of equal length “t” from another outlier with a single interval of length “2t”. The speed profile of the latter case is smoother, having the s-Peaks less random. Based on this logic, we want to have an unbiased weighted measure of the non-exponential contributions to s-IPI. R is thus defined as the relative non-exponential contribution weighted by the interval value as,

1 $$R=\frac{\sum _{j}{n}_{j,out}\times sIP{{I}_{j}}^{2}}{\sum _{i}{n}_{i}\times sIP{I}_{i}}(j > 10).$$

We fix the histogram bin width at 2 frames (~8ms) that optimizes the exponential fits. The exponential fit was done based on interval values below 10 frames (~40ms). Here $sIP{I}_{i}$ is the interval value in the i^th^ bin; *n*_*i*_ is the s-IPIs count in the i^th^ bin; ${n}_{j,out}$ is the s-IPIs count away from the exponential fit in the j^th^ bin. R has the same units as the s-IPIs (we take as the unit of sampling as 1 frame = 1/240 s to help visualize the result in this paper). Theoretically, R is proportional to the non-exponential ratio as well as the mean value of the non-exponential distributions (details given in the Supplementary Material). Of importance is that we have normalized R to be independent of the actual number of trials included in the experiments.

### Extra Validation of the R-parameter Definition via Computational Model Simulations

**Figure 4:**
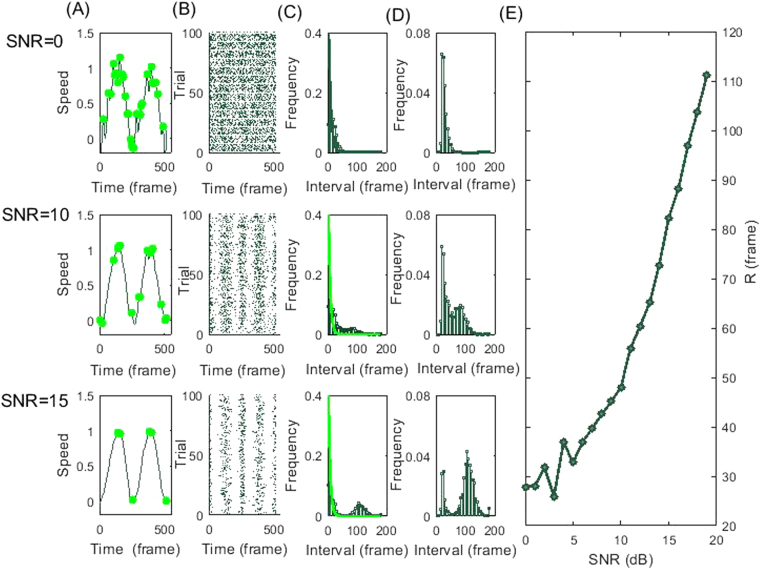
Simulation results quantifying speed fluctuations properties Column (**A**) plots simulated speed profiles with SNR = 0 dB, 10 dB, and 15 dB, respectively. Local s-Peaks are marked with green dots in each case. Column (**B**) shows the corresponding simulated raster-grams-like s-Peaks matrix (similar as in Fig. 2). Column (**C**) shows the corresponding Inter-Peak Intervals (IPI) frequency histograms (bin size set at 2 frames). Exponential curves were fit, with s-IPI interval values below 10 frames. Column (**D**) plots relative frequency histograms of the residual s-IPIs values outside the exponential fit region, for the three simulated cases. (**E**) plots the R calculated from the interval frequency histogram as a function of the simulated SNR. The parameter grows monotonically with SNR.

To further validate the definition of the R parameter, we tested it via numerical model simulations. We simulated the speed profiles for repeated forward-and-backward cycles with a “bell-shaped” curve^33^. Next, we added noise to the simulated signals with varying power. Figure 4(A) shows that, by systematically varying the *signal to noise ratio* (SNR) in the simulations, we obtained speed profiles having similar features as those we found in the experimental data, shown in Fig. 1. Numerically we reproduced similar s-Peaks patterns with similar s-IPIs distributions as shown in Figs 2 and 3. Figure 4(E) plots the calculated R-value versus the SNR used in the simulations. In the region tested, the R-parameter changes monotonically with the SNR in the simulations. This shows that the R-parameter very well characterizes the simulated speed profile’s SNR, i.e. the appearance of hidden speed fluctuations during motion.

After the validation of the R-parameter’s definition, we proceeded to apply it to the available experimental data. We will show below that it has an extraordinary individualized classification power in neurodevelopment.

### The R-parameter differentiates ASD and mature TD

**Figure 5:**
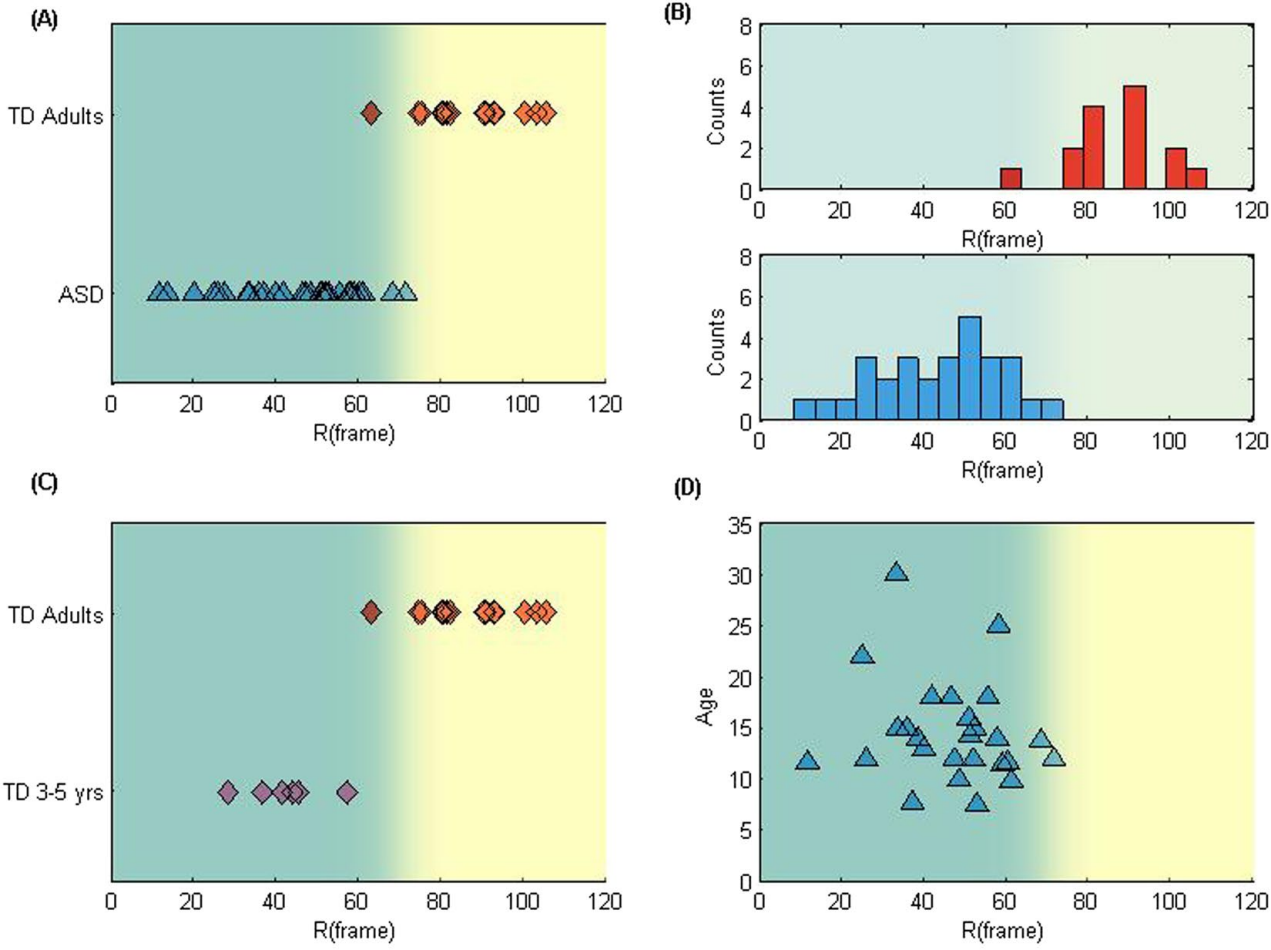
R-parameter captures significant difference between ASD and mature TD. (**A**) Location of each subject in the ASD group (29 subjects) and the mature TD group (15 subjects) on the R-space (x-axis is the R-parameter value). The space was colored used the logistic regression result $H(R)=g(0.30R-20.89)$, g is sigmoid function). (**B**) Distribution of R-parameter values among each group. The separation of the two groups is significant, with t-test ‘p’ value close to zero (below 10^−11^). (**C**) Shows a remarkable age-related maturation transition in typical development: here we compare R-parameter value results for 6 young TD children (3–5 years old) to 15 mature TD adults (19–31 years old). (**D**) Shows R-parameter results vs subject ages in the ASD group: notice the absence of an age correlated transition, concentrating all the R values to the lower ASD region. 26 subjects were included in the plot, the other 3 subjects’ ages (above 14 yrs) were missing in the records. The correlation coefficient between R and age in this group is −0.17, with p value equals 0.40.

We represent (Fig. 5A) all subjects with ASD and mature TD adults in the cohort along a one-dimensional R-phase space based on their calculated R-parameter values. The R-parameter analysis makes evident, at an individualized level, the clear distinction between ASD individuals and mature TD (Fig. 5(A,B)). The T-test p value was close to zero (below 10^−11^). Notice that these consistent differences among subjects were not obvious by just observing the motion with the naked eye. We have also tested that this distinction was robust to changes in the smoothing parameter selection (evidence provided in Supplementary Material). We computed a logistic regression model to separate ASD vs. TD R-space regions (colored accordingly) (details found in Method). The data set classification accuracy is 95.45%. Similar accuracy (above 90%) was obtained when we chose 70% of the data as training data with the remaining data as test set (details in Methods).

### The R-parameter captures TD neurodevelopmental trajectories

Being able to carry out efficient reaching movements is a result of motor development. Previous results suggest that typical motor development happens between 5–7 and 7–10 years old, as reviewed in Elliott *et al*.^18^. To test the power of the R-parameter in capturing motor developmental changes, we studied 6 TD children before their developmental period (3–5 yrs), and compared them to 15 mature TDs. Figure 5C shows a clear separation between immature and mature TD individuals in the R-space. This result shows that the R-parameter biomarker could characterize changes in typical motor development: It may also be used in future studies to illustrate the process in-between immature and mature neuro-development stages capturing the dynamic changes for everyone, including TD and NNDs.

### ASD R-parameter neurodevelopmental results

Comparing the ASD results in Fig. 5A and C, covering a broad age-range (7–30), all remained in the lower R region, at similar locations as young TD children. In Fig. 5D we further show that there is no age-related correlation in the R-parameter for this ASD cohort. Thus, we conjecture that for age above 7 years old there is no consistent age-related neurodevelopmental transition in the ASD group: They remained in the low R ASD region.

Note that the R-parameter appears to have a minimum threshold value found in our simulation results shown in Fig. 4E. For the representative subject denoted as ASD Case 1, the s-Peaks appear totally random (Fig. 2A), with the corresponding s-IPI distribution close to an exponential (Fig. 3A). The R-parameter value for this case is 24.9, suggesting also an experimental minimum threshold for R. Notice that some subjects in the ASD group have R values below 30 for ages above 10, even for the 30-years old ASD subject. This remarkable result suggests a total absence of age-related neurodevelopmental milestones in these ASD cases.

We further conjecture that the R-parameter biomarker may provide a powerful tool to quantitatively study and compare developmental processes across individuals. Studying other individuals in future could further test this hypothesis. The R-parameter measurements may provide an individualized neurodevelopment assessment, being potentially a tool for early detection of milestone deviations plus evaluations of longitudinal treatment responses.

We also found that the patients’ neurodevelopmental stages, as characterized by R, are well correlated with their psychiatric testing scores instead of their ages. This emphasizes that age might not be an effective mark to characterize neurodevelopment in ASD. Grouping individuals with ASD by age, as it is usually done, may blur important information about their maturity milestones, possibility affecting their clinical therapies.

### The R-parameter provides a quantatitive classification of ASD

Figure 5A, shows that the R-parameter has the power to separate ASD from mature TD. We further asked the question whether the R-parameter values may quantitatively characterize individualized functioning levels in the spectrum. Previous studies have pointed out correlations between motor ability and daily living skills^34^. They suggested a possible motor connection of ASD abnormalities with core autism characteristics^35^. However, previous attempts to use motor impairment as a possible diagnostic tool to differentiate subjects within the Autism spectrum have not been successful. A kinematic study^36^ showed some intriguing results about the differences in reaching-to-grasp motions between low and high/normal ability (based on IQ) in ASD individuals. However, those differences were not consistent when compared to a TD cohort group.

**Figure 6:**
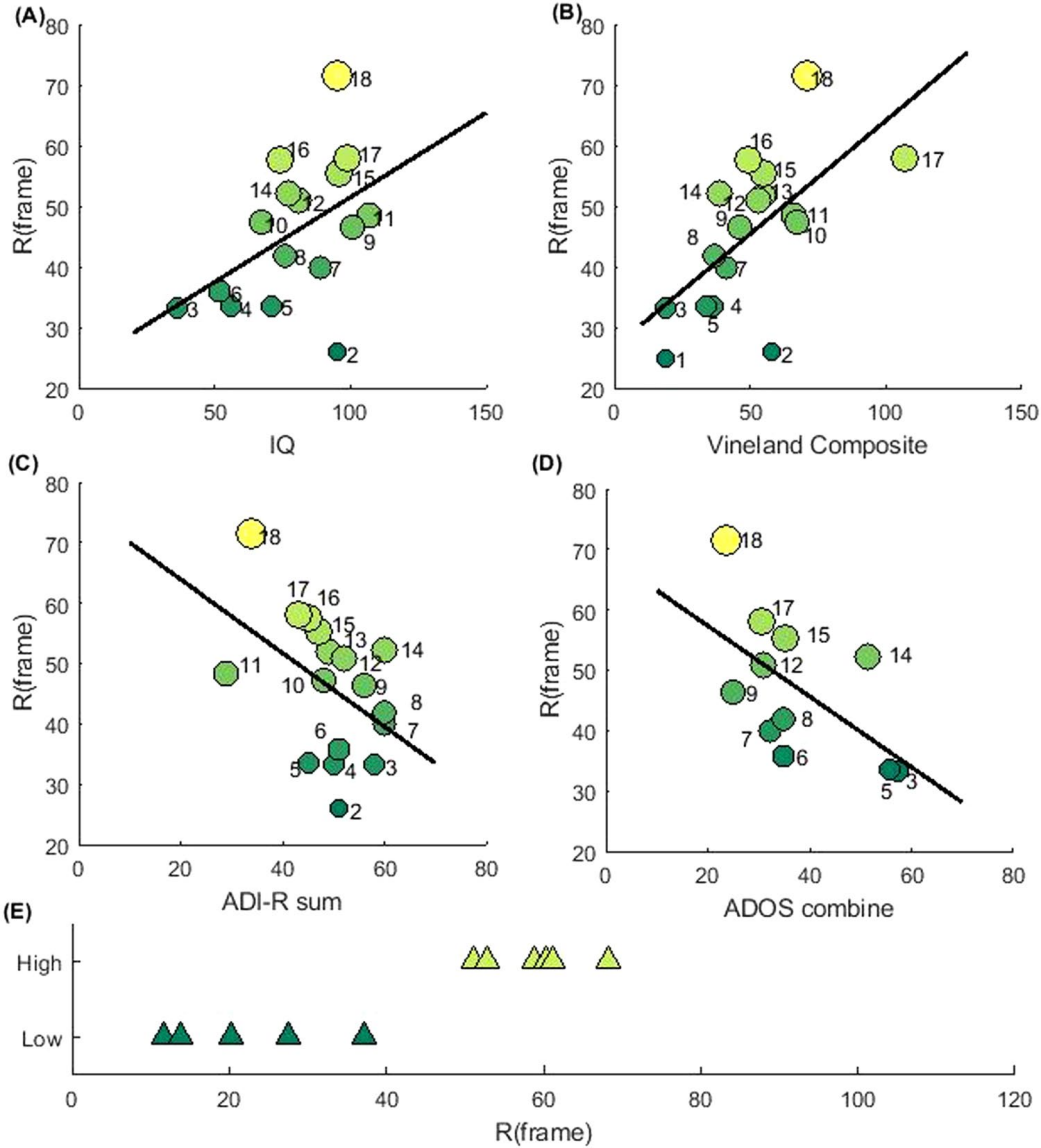
The R-parameter quantitatively characterizes ASD severity. Plots (**A**–**D**) of R-parameter vs. psychiatric test scores. (**A**) R-parameter is possitively correlated with IQ score (16 subjects). Data points are labeled by the subject index according to their R value rank. The black line denotes a linear regression fit. The significance p value for the Pearson Correlation between the R-parameter and IQ is 0.0680 (0.0104 if excluding the outlier subject #2 in the plot.) The significance p value for Kendal Rank correlation between R-parameter and IQ is 0.0714 (0.0208 if excluding subject #2). (**B**) The R-parameter is possitively correlated with Vineland Adaptive Behavior Composite score (17 subjects). The significance p value for the correlation between the R-parameter and Vineland Composite Score is 0.0064 for Pearson Correlation and 0.0044 for Kendal Rank Correlation. (**C**) R-parameter is negatively correlated with ADI-R sum scores (17 subjects). The significance p value for the correlation between R-parameter and ADI-R sum score is 0.0663 for Pearson Correlation (0.0346 if excluding subject #14) and 0.0569 for Kendal Rank Correlation (0. 0189 if excluding subject #14). (**D**) The R parameter is negatively correlated with ADOS combined score in module 2–4 (11 subjects). The ADOS combined score is calculated as a linear combination of ADOS Sect. B-D scores and ADOS module number. The weights were obtained from the Canonical Correlation analysis: 2*SectB* + 11*SectC* + 9*SectD*-5 *ModuleNumber*. The significance p value for the Kendal Rank Correlation between R-parameter and the ADOS combined number is 0.0264. (**E**) Further validation comes from including ASD subjects with labels given by the schools as low functioning or high functioning: a clear separation is found between high functioning (6 subjects) from ASD functioning (8 subjects).

Here, we instead compare the R-parameter values to the psychiatric test scores results in our ASD group. 18 subjects in our cohort had a battery of independent test results analyzed by a team of three psychiatrists: IQ (3-Leiter-R, the WISC-III or the WAIS tests), ADI-R, ADOS and Vineland. The IQ and Vineland tests provided functioning level measurements (i.e. ability): The ADI-R (a caregiver semi-structured interviewer assessment) and ADOS (an observational assessment) tests provided severity level assessments due to autistic symptoms (disability)^37,38^. Figure 6(A–D) provides strong support about the concurrence between our R-parameter results and these test scores. (1) Our R-parameter is significantly correlated (positive) with the functioning levels in the spectrum: the significance p-values for the Pearson Correlation and Kendall Rank Correlation between our R-parameter and Vineland Composite Score were both below 0.01; the significance p-values for Pearson Correlation and Kendall Rank Correlation between R and IQ were below 0.05, if we exlude one outlier subject in the comparison. (2) Our R-parameter is modestly correlated (negative) with ASD severity level in the spectrum. The p-values for the correlation between R-parameter and ADI-R sum score were slightly above 0.05 (below 0.05 when we exclude the outlier subject). We didn’t find, however, a direct correlation between our R parameter and the ADOS score. Through canconical correlation analysis, we found a negative correlation (p value below 0.05) between R-parameter and a combined ADOS test scores: 2*SectB* + 11*SectC* + 9*SectD*-5*ModuleNumber* in module 2–4. Future studies with a larger cohort may be neecessary to test the relation between R and ADOS scores All in all, our R-parameter is positively correlated with functioining levels and negatively correlated with ASD severity.

For further validation and testing, we included the remaining 11 ASD subjects that were labeled in the school as high functioning or low functioning. We found that in the R-space the 6 subjects labeled high-functioning were clearly separated from the remaining 5 subjects that were labeled low-functioning (Fig. 6D). This result provides added evidence about the independent reliability of using the R-parameter classification.

Our results have thus unraveled a significant correlation between movement and other aspects of Autism development. The R-motion feature captured here, i.e. the noise level present in the motor output, may provide an underlying mechanism contributing to the variable motion disturbances present in the spectrum, as well as in other autistic symptons.

### R-parameter uncovers possible ASD familial link

**Figure 7:**
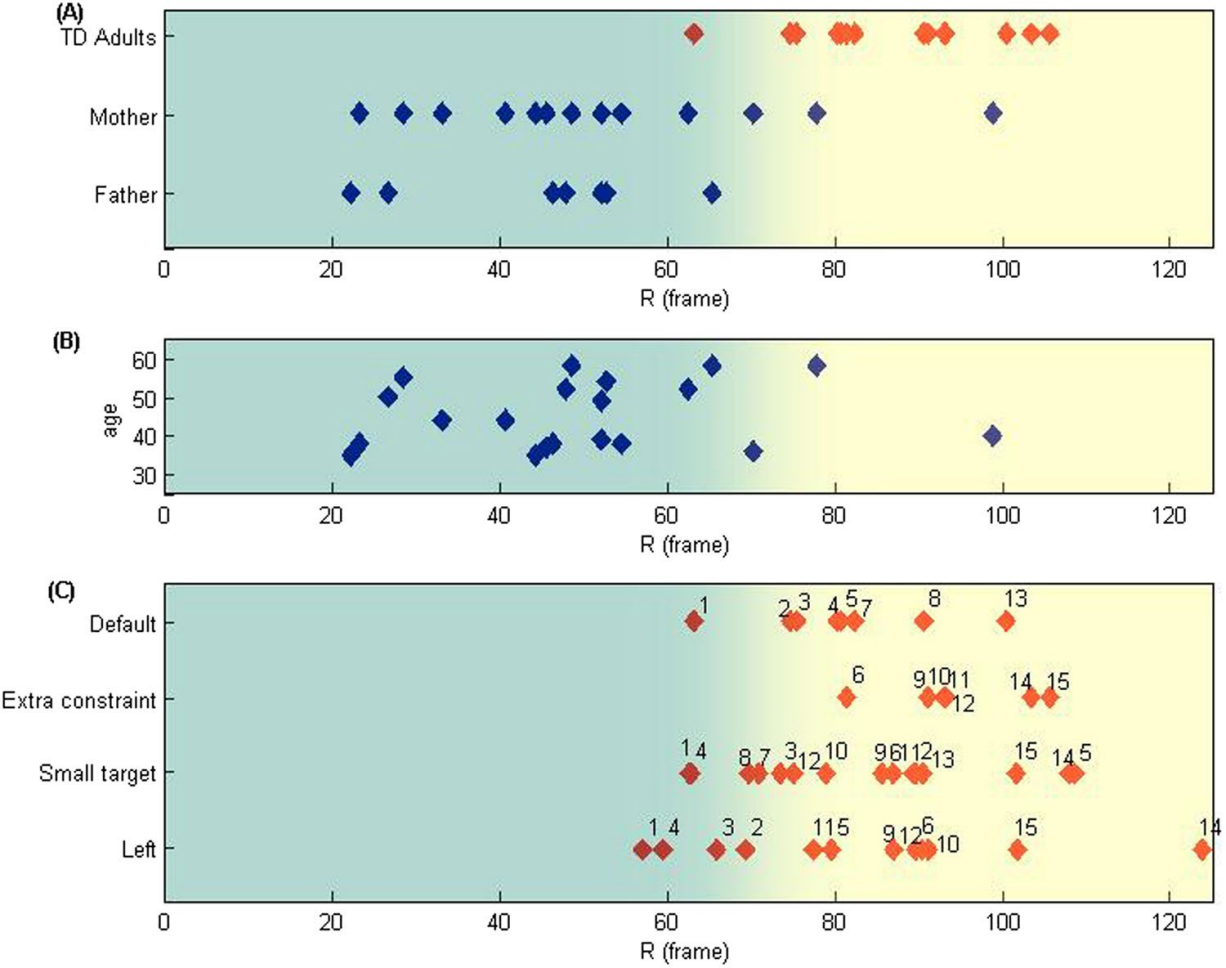
Filial ASD link and further R-parameter robustness validation (A) R-parameter results from measurements in 20 parents of 13 ASD subjects in our cohort compared to typical control adults. Most parents (17 out of 20, with 10 mothers and 7 fathers) fell within the ASD region on the R-space. They are clearly separated from typical control individuals. (**B**) Parent’s age vs. R-values: no correlation is found. Having correlation coefficient 0.15 and p value 0.53. (**C**) TD control variation R-parameter test when varying the experimental conditions: (1) by changing the trajectory (adding restriction to the motion trajectory) (test on 7 subjects); (2) by changing target sizes (from 5 cm to 1 cm) (test on 15 subjects); (3) left-hand testing (all subjects were right handed) (test on 12 subjects). Data points were labeled by their R-value rank against the default condition. Notice the consistence of the R values under various conditions. All subjects remain in or near to the TD region in R-space, as in the default condition, suggesting the robustness of our R-results. The R biomarker value captures individual’s motion features that are independent of task design.

In ASD studies, significant concordance reports show its high heritability: high recurrence in twins^39^ and families^40^, increasing risk with increased relatedness^41^. The high heterogeneity of ASD symptomatic behaviors, even in affected siblings^42^, impedes a clear identification of generic genetic filial links. Previous studies have reported an increased risk of having problems with communications and social interactions (known as “broad phenotype”) in non-diagnosed ASD relatives^43^. We decided to ask the question whether our R-parameter could provide extra information about potential familial links, which may contribute to future genetic studies. We thus included 20 parents of 13 ASD participants in our cohort. Figure 7A shows their R-parameter results: 17 out of 20 parents (10 out of 13 mothers, and all fathers) fell in the ASD R-region, having lower R-values than TD adults. ASD Parents available are generally older than the TD adults tested, aging from 30 to 60 years old. However, we tested there is no age-related correlation in the ASD parents’ R-values (correlation coefficient equals 0.15, p = 0.53) (shown in Fig. 7B). Therefore, we conclude that aging is not the reason for their deviation from the TD adults on the R-plane. We suggest that there may be certain “atypical” neurodevelopment in these parents which might contain a neurodevelopmental link with their ASD diagnosed children. The movement R-biomarker unveiled in this paper may help provide another potential familial links in ASD, as well as in other neurological disorders, which may help unravel genetic connections in future studies.

### Further R-parameter robustness validation

A valid biomarker should robustly capture traits that should separate atypical developments from normal variations in typical development. It should also be inherent to the nervous system development and invariant to certain external effects. We have further justified the R-parameter robustness by testing it against variations in the experiment setup conditions. We tested typical controls under the following experimental changes (see details in Supplementary Information): a) by changing motion trajectory/arm configurations introducing task restrictions (test on 7 subjects); b) increasing task difficulty level (test on 15 subjects); c) testing with the non-dominant left hand (test on 12 subjects). We found that the R-parameter values remain the same and are consistent against all these changes, remaining in the same R-parameter TD region as in the default experimental condition (Fig. 7C). This implies that the distinctions across subjects, if any, due to their motion trajectories, task difficulty level or proficiency level doesn’t change the R-parameter classifications we found. We believe that the motion features captured by R are fundamentally intrinsic to everyone’s nervous system development.

## Discussion

The work presented here was aimed at developing a data-driven approach to provide objective behavioral neurodevelopmental assessments for NDD, to contribute at narrowing down the present wide gap between clinical behavioral observations and neuroscience studies.

To achieve this goal, we unraveled a new physiological data set that quantifies the noise levels present in the continuous movement outputs. Movement (3D positional trajectories) was tracked by using high-resolution electromagnetic sensors at 240 Hz, providing data not evident to the naked eye. We separated spurious electric noise from the measurements preserving the inherent movement output noise using a carefully designed smoothing algorithm. We extracted hidden motion fluctuations, at millisecond time scales, from the continuous speed profiles. Using a novel statistical analysis of the hidden fluctuation, we derived a putative biomarker, uncovering important physiological information present in the movement output. Applying our metrics in the cohort we studied showed a high classification power to characterize individuals’ neurodevelopment stages.

First, our biomarker clearly captures the deviations of ASD systems from mature TD systems, independent of ages. Second, it provides a quantitative classification that represents functioning levels and ASD severity in the spectrum, with explicit agreement with a battery of psychiatric clinical tests. Third, it characterizes the neurodevelopmental milestone trajectories in typical development by comparing systems before and after motion maturity. Fourth, it captures a surprising familial motor link associated with the ASD disorder.

The biomarker proposed here overcomes some of the limitations present in recent studies using machine learning techniques^9^. The selection of our parameters is not random, but hypothesis-driven and biologically meaningful. Our result goes beyond providing patient vs. control classifications. It provides maladies severity levels with higher degree of accuracy than previous behavioral classifications^44,45^. Moreover, it also has the following advantages for making it a putative robust nervous system development biomarker: (1) It identifies atypical disorder traits that are clearly separated from variations present in normative data of TD individuals. (2) It is precise enough to apply to each and all individuals. (3) It unravels fundamental distinctions across individuals in their neurodevelopment. These distinctions are not detectable by traditional visual observations, nor do they originate from certain traditionally used motion features: e.g. average speed, cycle duration, motion trajectory, task difficulty and motion proficiency.

Our remarkable results emphasize the importance of the speed fluctuations in human motions, when looked at millisecond time scales. It makes evident that there is a wealth of physiological information hidden in our natural motions when looked at higher temporal precision. Compared to traditional kinematic analyses, the motion feature we captured here is robust and fundamental to the motor system. It may provide insight to the study of motor control and developmental theories, as well as providing an underlying mechanism to observations in previous studies. Further studies are needed to understand how TD individuals develop compensatory strategies to control their internally produced noises as they mature and how such mechanisms are disrupted in ASD or other NDD individuals. Our findings may potentially provide a promising direction for future ASD etiology research, as well as an extra objective guidance for future genetic and pathological neuroscience studies.

Future application of the metrics described here to larger cohorts of subjects or in longitudinal studies may provide a more precise neurodevelopmental map, by using the R-parameter as well as its rate of change. This map would be a powerful added tool to study individualized neurodevelopmental process and the deviations in ASD and other NNDs. It may also capture subtle changes occurring over time that maybe missing from the present static diagnostic tools. It may have the potential of assisting effective predictions of treatment responses in the future. Comparing to the psychiatric clinical tests, our biomarker also reports results from normative data on TD systems, making it an objective supplementary tool to neurodevelopment assessments. The motion statistical analyses developed here can also be potentially applied to other types of human disorders, as well as in animal models, to check on the generality of the results on ASD presented here.

In summary, we introduced in this paper a putative biomarker quantifying hidden motion fluctuations during simple natural reaching motions. The biomarker enables a precise and robust characterization of individualized neurodevelopment. Application of this biomarker to the available cohort unraveled remarkable physiological information: an automatic ASD vs. TD distinction, a severity ASD quantification, age development landmarks in TD, as well as potential familial link associated with ASD. It has the potential to be a powerful tool to generally study neurodevelopment as well as other NNDs.

## Methods

### Subjects

The cohort studied here included 71 individuals: 30 ASD subjects with a 7–30 broad age range; 15 subjects were typically developing (TD) adults (19–31 years old); 6 were TD 3–5-years old children; 20 were the ASD parents participating in our studies (who did not have an ASD diagnostic). Psychiatrists as well as qualified professional clinicians or agencies provided the ASD diagnoses. Among the ASD subjects, 18 were provided with complete psychiatric test by four scores (IQ, Vineland Adaptive Behavior Composite score, ADI-R, ADOS). The diagnoses were agreed by three psychiatrists, including one of us (JIN). The remaining 12 subjects were diagnosed and labeled as high functioning or low functioning by certified clinicians. The participant’s demographic information is provided in Supplementary Tables 1–4. We reported the individualized results in the main body of the paper. One ASD subject’s data was excluded from the discussion because of the high electric noise present in this case (details given in Supplementary Material).

### Experimental Design

All subjects performed the basic pointing task paradigm as illustrated in Fig. 1A. They sat comfortably in front of a touch screen, pointing to a target (diameter 5 cm) at the center of the screen and retracted their hand (right hand, dominant hand) naturally after pointing. They moved at their own comfortable pace. The target disappeared after it was touched, later reappearing to initiate the next reach. The experimental setting was changed for 7 out of 15 TD adults (randomly selected) to introduce some variability in the motion trajectories: the arm movements were constricted to be above the table in the new setting (see Supplementary Information for details). Typical control adults did additional tests with decreased target size (1 cm), as well as tests done using the left hand, to test the robustness of the results further. Their hand motions were continuously captured at 240 Hz using electromagnetic sensors (Polhemus Liberty, Colchester, VT). Protocols were approved by the Institutional Review Boards at Rutgers University and Indiana University, in compliance with the Helsinki Declaration. All subjects provided informed consent for participation in the study. Subjects under 18 provided assent and their parents/guardians provided consent. All experiments were performed in accordance with relevant guidelines and regulations at Rutgers University and Indiana University.

### Smoothing Algorithm

The electromagnetic sensor reports its position calculated from its electromagnetic signal, which is a combination of the “real” signal and the inevitable electromagnetic noise. A challenge was to separate such electromagnetic noise while preserving the signal information. To achieve this goal, we applied a triangular smoothing algorithm to the velocity profiles calculated from the recorded position data in each direction. The triangular smoothing algorithm was applied by using the following moving triangular window:

2 $$v\text{'}(i)=\frac{\sum _{k=-d}^{d}(v(k+i)\cdot (d+1-|k|))}{\sum _{k=-d}^{d}(d+1-|k|)}.$$

Here, $v(i)$ is the i^th^ element of the original speed velocity profile $v\text{'}(i)$ is the ith element of the smoothed profile, and k is the summation index, going from −d to +d. The number of elements in the sliding window was chosen here was 25 with d = 12 and k running from k = −12 to k = 12. This process builds up a symmetric weighted sum around the central point.

The filtering process is a convolution of the experimentally measured signal with the triangular filtering function. The triangular function can be written as:

3 $$g(t)=-\frac{|t|}{{\tau }^{2}}+\frac{1}{\tau },|t|\le \tau .$$

The filtered signal is: *S*_*f*_ (*t*) = *s*(t) * *g*(*t*), with *s*(*t*) denoting the raw empirical data signal. From the convolution theorem, the Fourier transform of the filtered data equals the product of the Fourier transform of the raw data times the Fourier transform of the triangular function: $F\{s\,\ast \,g\}=F\{s\}\cdot F\{g\}$. Hence, the frequency response of the triangular filtered data, namely the power spectral ratio between the filtered data and the raw data is

4 $${|F\{g\}(f)|}^{2}={|\hat{g}(f)|}^{2}=\frac{1}{{\pi }^{4}{f}^{4}{\tau }^{4}}{\sin }^{4}(\pi f\tau )\sim \frac{1}{{\pi }^{4}{\tau }^{4}}{f}^{-4}.$$

The response at 20 Hz is <−20dB, while at 60 Hz is <−39dB. Notice that the application of the smoothing algorithm to the velocity profiles is equivalent to the application to the raw positional readings directly.

We provide justification evidence for using the triangular smoothing algorithm in the Supplementary Material. We illustrated also there that the smoothing algorithm is sufficient to filter out external electromagnetic noise as well as preserving temporal information of the local fluctuations in the velocity profiles. We also provide justification for our smoothing parameter selection as well as support for the robustness of our results in the Supplementary Material.

### Cycle selection

We identified motions during forward-and-backward cycles, only including s-Peaks occurring inside these cycles but not the fluctuations occurring when the hand was at rest in the lap. The criteria used when selecting forward-and-backward motion cycles followed the processes: (a) Screen touch points were identified from the positional trajectories in the direction perpendicular to the screen. (1) Identified peaks in the position trajectories; (2) Selected peaks with the position above the mean plus one standard deviation for the whole process; (3) The period between two adjacent peaks was above 1.25 s (300 frames). (b) Start and stop cycle points were identified from the speed profiles in-between adjacent touch points: (1) Start/stop points were identified as points when the velocity in the direction perpendicular to the screen changed direction; (2) Start/stop points were at least 0.42 s (100 frames) away from the touch points; (3) Start/stop points’ positional displacement were below the 1/3 of the maximum displacement in between the touch points; (4) Speed at start/stop points were smaller than the average speed; (5) select the points nearest to the touch points satisfying the above criteria (6) Delete outliers: Temporal duration of the cycle is above 2.5 s (600 frames) or the maximum speed during the cycle is below 0.2 m/s. We validated the cycle identifications by visually checking the corresponding speed profiles and positional trajectories.

### Logistic Regression

We applied logistic regression to the datasets including all ASD subjects and TD adults, using the MATLAB built in function *fminunc* (Matlab and Statistics Toolbox Release 2012a, The MathWorks, Inc. Natick, Massachusetts, United States). The regression result is $H(R)=g(0.30R-20.89)$, with $g(z)=\frac{1}{1+{e}^{-z}}$, the sigmoid function. H(R) gives the possibility of being a mature TD individual. The middle point separating the two regions is at the 69.47 frame. The classification accuracy of this model in the dataset is 95.45%.

To test the robustness of this result, we randomly took 70% of the dataset as a new training data set using the remaining 30% as the test set. The average training set accuracy (in 50 iterations) is 95.73% and the average test set accuracy is 97.86%.

### Research objectives

After the initial analyses of the data, we conjectured that the hidden speed fluctuations we unraveled could contain important physiological information about neurodevelopment. We proceeded to test this hypothesis by identifying a single parameter that quantifies the statistical fluctuations of the physiological components in the data. We further justified this definition through a theoretical computational model. We then tested the relevance of such a parameter against the measured experimental data. We found an ‘*a posterior*’ agreement between the parameter values we determined from the data and a battery of clinical diagnosed neurodevelopment stages carried out by psychiatrists.

### Blinding

We applied the same quantitative algorithms, blindly, without knowing at all to which subjects each data set belonged to. After doing our blind statistical analyses, we found a very close correlation between the psychiatric diagnoses and our results.

### Outliers

The data of one subject, out of 72, in our cohort showed higher external noise levels than the others. We excluded this subject in the discussion but provided detail information about it in the Supplementary Material.

### Pre-processing

We started by collecting the raw data using wearable electromagnetic sensors. We then applied a triangular smoothing algorithm to the raw data calculated velocity profiles. The justification for using this smoothing algorithm and the selection of the the smoothing parameter values is provided in the Results and in the Supplementary Material.

### Statistics

It is important to emphasize that we are not considering a comparative group statistical analysis, as it is traditionally done in group studies. Instead, we studied each individual subject independently each time in their own right. We may call our results for each subject their own movement “DNA”. The correlations, found between our automatic emergent individualized classifications vs. the clinical diagnoses, were assessed by the classification accuracy, i.e. the percentage of correctly classified samples out of the total. We found over 90% accuracy when compared to their clinical diagnoses.

### Simulations

To test the validity of our approach we numerically simulated a theoretical model of speed profiles for repeated cycles with various noise levels. In each calculation, we simulated 300 forward-and-backward cycles. The cycles time duration was fixed at 480 frames (240 frames for forward-and-backward processes, respectively). The speed for each forward (or backward) process was simulated as a bell-shaped curve within a minimum jerk model^33^ plus noise:

5 $$v(t)={(4\tau (1-\tau ))}^{2},\tau =t/{T}_{1}\,{\rm{for}}\,{t} < {T}_{1},\tau =(t-{T}_{1})/{T}_{2}\,{\rm{for}}\,{T}_{1} < t < T.$$

As found in the experiments, the bell shape speed profiles we used were asymmetric. We set T = T1 + T2 = 240, T1 is the time duration before reaching the maximum speed peak and T2 is the time duration after the peak. Resting periods were added before and after each cycle, as well as in-between the forward and backward processes, with 20 frames of length. We added white Gaussian noise homogeneously during the whole process with different signal-to-noise ratios.

### S-Peaks vector and matrix

The s-Peaks cycle vectors were defined by

6 $${s}_{i}(j)=\{\begin{array}{c}1,\,{\rm{one}}\,{\rm{or}}\,{\rm{more}}\,s\mathrm{Peaks}\,{\rm{occurs}}\,{\rm{at}}\,{{\rm{j}}}^{th}\,{\rm{sampling}}\,{\rm{point}}\\ 0,\,{\rm{no}}\,s\mathrm{Peak}\,{\rm{at}}\,{{\rm{j}}}^{th}\,{\rm{sampling}}\,{\rm{point}}\end{array},\,(j=1,2,\mathrm{...}{,N}_{j})\,{\rm{for}}\,{{\rm{i}}}^{th}\,{\rm{cycle}}$$

The components of the s-Peak matrix are $M(i,j)={s}_{i}(j)$ (i = 1, 2, …, *N*_*i*_; j = 1, 2, …, *N*_*j*_); *N*_*i*_ is the number of full forward and backward cycles (usually 100 cycles); *N*_*j*_ is the number of frames in each cycle. To simplify visualization in Fig. 2, we took *N*_*j*_ as 800 (~3.4 s), with 400 frames (~1.7 s) before and after the touching points for all subjects. In all the calculations described here, *N*_*j*_ was chosen as the mean cycle length value across repetitions for each subject.

### Data Availability

The datasets generated during and/or analyzed during the current study are available from the corresponding author upon reasonable requests.

## Electronic supplementary material

Supplementary Material

Animation
