# Supplementary material for: A Biomarker Characterizing Neurodevelopment with applications in Autism
Source: Sci Rep. 2018 Jan 12;8:614. doi: 10.1038/s41598-017-18902-w (PMC5766517; doi:10.1038/s41598-017-18902-w)
Supplement: Supplementary file 1 — Supplementary Material [file 41598_2017_18902_MOESM1_ESM.pdf]

Supplementary Material SREP-17-31320A

A Biomarker Characterizing Neurodevelopment with applications in Autism

Di Wu^{1,4,†}, Jorge V. José^{1-4,†, ‡,*}, John I. Nurnberger⁵ and Elizabeth B. Torres⁶⁻⁸

¹Physics Department, Indiana University, Bloomington, Indiana.

²Stark Neuroscience Institute, Indiana University School of Medicine, Indianapolis.

³Department of Cellular and Integrative Physiology, Indiana University School of Medicine, Indianapolis.

⁴Key Laboratory of Theoretical Physics, Institute of Theoretical Physics, Chinese Academy of Sciences, Beijing, China.

⁵Institute of Psychiatric Research, Department of Psychiatry, Indiana University School of Medicine, Indianapolis.

⁶Psychology Department, Rutgers University, New Brunswick, New Jersey.

⁷Rutgers Center for Cognitive Science, Rutgers University, New Brunswick, New Jersey.

⁸Center for Biomedical Imaging and Modeling, Computer Science Department, Rutgers University, New Brunswick, New Jersey.

†, ‡ permanent address,

*corresponding author: jjosev@iu.edu

Triangular smoothing algorithm

The triangular smoothing algorithm was used to filter out external noise in the velocity profiles by using the following moving triangular window (as shown in Fig. S1(A)):

$$v'(i) = \frac{\sum_{k=-d}^d (v(k+i) \cdot (d+1-|k|))}{\sum_{k=-d}^d (d+1-|k|)}.$$

Here, $v(i)$ is the ith element of the original speed velocity profile $v'(i)$ is the ith element of the smoothed profile, and k is the summation index, going from $-d$ to $+d$. In this paper, the number of elements in the sliding window was chosen as 25 with $d = 12$ and k running from $k = -12$ to $k = 12$. This process builds up a symmetric weighted sum around the central point. We will illustrate below that this algorithm is sufficient to filter out external electromagnetic noise as well as preserving temporal information of the local fluctuations in the velocity profiles. We will also provide justification for our smoothing parameter selection as well as support for the robustness of our results.

1. The smoothing algorithm preserves local peaks temporal information

As shown in Fig. S1(B), the advantage of using the triangular smoothing algorithm is its preservation of the local peaks' temporal positions. We provided more evidence in Fig. S2, where we compared results from applying a triangular smoothing algorithm (smoothing window width=104ms, 25 frames), a rectangular smoothing algorithm (smoothing window width=104ms, 25 frames) and a Butterworth Low Pass filter (6th order, cutoff frequency=10Hz) on a simulated signal (sampling rate=240Hz). The signal was simulated as $s = \sin(2\pi t / 500) + \text{whitenoise}$. As shown in the Fig. S2, compared to rectangular smoothing algorithm, the triangular smoothing algorithm captures the appearance of the low frequency fluctuations. Compared to the Butterworth Lowpass filter, the triangular smoothing algorithm keeps better the temporal position of the fluctuations. The plot in Fig. S1(C) provides an example of the filtering effect on the experimental data. The filtered data captures well the temporal information of the original velocity profiles. Fig. S1(D) shows the corresponding speed profiles calculated from the filtered velocity profiles in all three directions.

2. The smoothing algorithm is sufficient to filter the external electromagnetic noise

The triangular smoothing algorithm is applied in the temporal domain. Its frequency response is illustrated in Fig. S1(E-F) with both theoretical and experimental data. In the frequency domain, it is a low pass filter. In the Method section of the main paper, we have showed theoretically the selected smoothing parameter is sufficient to eliminate the high frequency electromagnetic noise in the data collected. Below we provide numerical simulation and experimental evidence supporting this statement.

Numerical analysis: To further test our approach we generated a signal with constant power between -120 to 120 Hz:

using $y = \sin c(\pi t) = \frac{\sin(\pi t)}{\pi t}$, with sampling at 240Hz. We applied our triangular filtering algorithm to the numerically generated data, calculating the power spectral ratio between the filtered and original signals (the frequency response). As shown in FigS.1(C), this curve ratios agrees very well when compared to the theoretical result.

Experimental data: Next we analyzed frequency response of our filtering approach by testing it on data collected during experiments. FigS. 1(D) plots the power spectra density of the collected raw data (1min in one case of ASD) as well as the filtered data. The noise above 20Hz was clearly filtered out after using the triangular filtering algorithm.

3. No differences found in the external electromagnetic noise

No filter could guarantee perfectly filtering out all possible external noises while keeping the ‘real’ signal. Even if there might be noise leftover in the filtered data, we will show below that the extrenous external noise did not contribute to significant changes in the ASD group separations discussed in the paper. We tested this by quantifying the noise levels in the velocity profile in the direction perpendicular to the screen. The noise levels were calculated for each subject with data collected in the first second (resting phase). Squared root average of the differences between raw velocity and filtered velocity data was calculated. As shown in FigS. 1(G), there is no significant difference between the ASD group and typical control adults (T-test p value equals 0.42). There is one case with significant higher noise levels. The experimental conditions might not be well controlled during this experiment. This subject is considered as an outlier and excluded from the discussion. FigS. 1(H) further illustrates that there is no correlation between external noise power and the calculated R value (correlation p value equals 0.19, excluding the outlier). Hence, there is no significant external electromagnetic noise contribution in the group clusters distinctions in our results.

All in all, our triangular smoothing algorithm is sufficient to filter out the external electromagnetic noise as well as to preserve the local peaks temporal information and structure in the velocity signals.

Theortical R-parameter analysis

R could be written as $R = \frac{\sum_i r_i^2}{\sum_i x_i + \sum_i r_i}$, x_i are the intervals falling into the exponential distribution, and r_i are the

intervals falling outside of the exponential distribution. Take α as the non-exponential distribution ratio, i.e.

$\alpha = \frac{\sum_i r_i}{\sum_i x_i + \sum_i r_i}$. Then $R = \alpha \frac{\sum_i r_i^2}{\sum_i r_i}$. Assuming r_i is gaussian distributed with average equals m and variance equals

σ^2 . $R = \alpha \frac{\sigma^2 + m^2}{m} \propto \alpha m$. Therefore, R is propotional to the non-exponential distribution ratio, and the mean value of the non-exponential distribution.

Robustness of R-classification to changes in the smoothing parameter selection

Identification of the s-Peaks depends on the value of the smoothing parameter selected. We found, however, that the distinction in s-IPI statistics between ASD and TD groups remains robust for a broad range of smoothing parameter selections. FigS. 3C shows that, when the smoothing window width is minuscule, the present fluctuations are mainly from external electromagnetic noise. The temporal dynamics (R-value) for such external noise are not separable between individuals. As the smoothing power (smoothing window width) increases, the external electromagnetic noise was gradually filtered out, and the distinction in the signals started to become evident. We chose the smoothing window width at 25 frames (104ms) to guarantee that the separation among subgroups was clear and that the R-parameter values had not yet saturated.

To further illustrate the robustness of the main results of the paper about ASD subjects’ distinctions, we doubled the smoothing window width (to 49 frames instead of 25). FigS. 3 (A-B) compare the filtered velocity profiles together with the corresponding speed profiles under the two smoothing windows width selections. By comparison, the doubled smoothing window width selection (49 frames) possibly missed some local peaks in the speed profile. However, the group distinction between ASD group and Control group is still significant (FigS. 3D).

R-value robustness to variations in experimental design

1). **The subjects' cluster R-value classifications is independent of motion trajectories/arm configurations.** We did not impose any restrictions during the experiments on how the subjects moved: subjects reached and retracted their arms as they wish. There might be, however, arm configuration/motion trajectory differences across subjects. We tested the possible effects of the arm configuration/motion trajectory on the R-value on 7 out of the 15 typical control adults. We used a different experimental set up.

As shown in Fig. S3(A-B), in the default experimental setting (condition 1), subjects were totally free to move their arms and hands. Their hands rested on their laps or on the arm of the chair during the resting period. When touching the screen, their arms did not necessarily have to be fully stretched. In condition 2 (Fig. S3(C-D)), the screen was moved farther away from the subject. The subjects were then asked to keep their arm above the table coming back to a fixed resting position on the table after reaching (the yellow dot). Under the latter condition, most subjects needed to fully stretch their arm to reach to the target point. In Fig. S3(E-H) we show a corresponding arm avatar for motions during the reaching and retracting cycle in two conditions (view from side(E,G) and view from above (F,H)). Blue dot is the position of sensor put in the upper arm, as an estimation of the position of elbow. By comparison of E and G, 1) there is more change in the elbow angle (angle between upper arm and lower arm) in condition 2 than condition 1; 2) the arm is fully stretched in condition 2, not in condition 1. Comparison of the horizontal plane trajectory (view from above, F and H) shows the difference of arm configuration under the two conditions. In Condition 1, the elbow is to the right of the right shoulder, i.e. to the outer space of the body. In condition 2 the elbow is to the left of the right shoulder, i.e. to the inner space of the body. Overall, arm motion under condition 1 is more relaxed, while arm motion under condition 2 is more restricted. Despite the changes introduced in the arm configurations, the R values are not separable under the two conditions (F).

This suggests that the R-biomarker is independent of the specific motion trajectory followed during the reaching-and-retracting task. Possible variations present in the motion trajectories, if any, across subjects did not contribute to R cluster distinctions.

2). **The subjects' R-value cluster classification is independent of the task difficulty level.** A given task may have different difficulty levels for different individuals. We questioned whether such difference across individuals may contribute to the subjects' R-value distinctions. To answer such concern, we increased the degree of task difficulty by decreasing the size of the target (diameter changed from 5cm to 1cm) testing TD adults under such change. As shown in Fig. 6B, all TD subjects remained in the same cluster region as in the default condition. We found no task difficulty changes in the R-values. The distinction between subjects still held, even as the task difficulty increased: the TD adults' R- values doing a harder task remained separated from those ASD subjects' R-values while doing an easier task.

3). The R-cluster classification is independent on the potential differences in motion proficiency level across subjects.

One could argue that typical control adults have smoother speed profiles simply because they have practiced more similar motions than others may in their lifetimes. One important fact answering this concern is our finding of the lack of age related R-value differentiation in ASD: just practicing more cannot explain the R-value differences. We further tested left-hand motions in TD control adults, which provides extra support. All subjects in the cohort studied are right handed. Their left-hand motions are supposed to be less proficient than their right-hand motions. As shown in Fig.6B, however, we did not find any consistent differences in the R-values from having left or right hand motions: Therefore, this supports the fact that R captures inherent individualized physiological features independent of left or right hand motions. Moreover, the R-value distinctions we found here are not simply a matter of proficiency, but they are fundamentally intrinsic to everyone's nervous system development.

Supplementary Figures

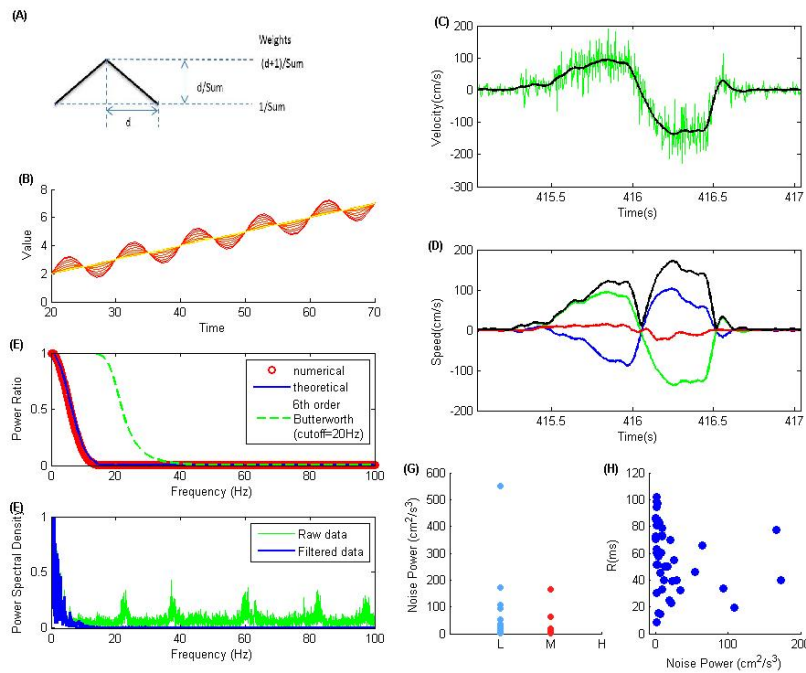

Fig. S1. Triangular smoothing algorithm with validation (A) The triangular smoothing algorithm was applied to the experimental data using a sliding window of width $2d+1$. Weights were distributed using a symmetrical triangle as shown in the figure. (B) Triangular smoothing applied to a simulated periodic data set ($y = \sin(\pi t / 5) + 0.1t$). The smoothing window size varied from 0-30 (red to yellow). The triangular smoothing preserves the location of the s-Peaks. (C) Smoothing effects in experimental data: one velocity profile example in one cycle in the direction perpendicular to

the screen. Green line: velocity profile calculated from raw positional data; black line: filtered velocity profile after applying the triangular smoothing algorithm (25 frames smoothing window width). (D) Filtered velocities along the three orthogonal directions (blue, green, red) . The corresponding speed profiles were calculated from the velocities (black). (E) Smoothing algorithm frequency response compared to traditional low pass Butterworth filter¹: The figure shows the power spectrum ratios between the filtered signal spectral power density (triangular filtering with window width of 25 frames) and the original data power spectral density as a function of frequency f . Blue denotes the theoretical curve; Red circles the numerical simulation results: The Green curve was obtained from a 6th order Butterworth filter with cutoff frequency at 20Hz. (F) Power spectral data density (filtered and unfiltered) from experiments (velocity in the direction perpendicular to the screen; duration: 1min; subject group: ASD): green line: before filtering; blue line: after filtering (triangular smoothing with 25 frames smoothing window). (G) Comparison of external noise power level across groups: No significant separation between ASD and TD groups (T test p value equals 0.42). The one subject with significant higher external noise level than the others is considered as an outlier because of possible special experiment condition differences. Noise was calculated as the squared difference between the raw velocity and filtered velocity in the direction perpendicular to the screen in the first second of data collected. (H) There is no experimental correlation between the mean s-IPI parameter and the external noise power during the experiment (correlation p value equals 0.19, excluding the outlier).

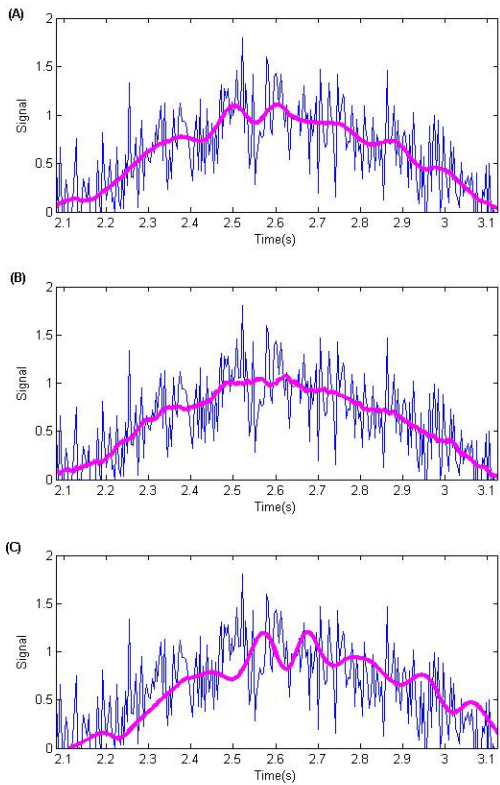

Fig. S2. Advantages of using Triangular Smoothing Algorithm Results from applying smoothing algorithm to a simulated signal: (A) Triangular Smoothing Algorithm (smoothing window width equals 25 frames, 104ms) (B) Rectangular Smoothing Algorithm (smoothing window width equals 25 frames, 104ms) (C) Butterworth Lowpass filter (6th order, cutoff frequency equals 10Hz). The simulated signal is $s = \sin(2\pi t / 500)$ (t is time in sampling frame) plus white noise. Blue lines denote the raw signal, and the thick purple lines denote the filtered signals.

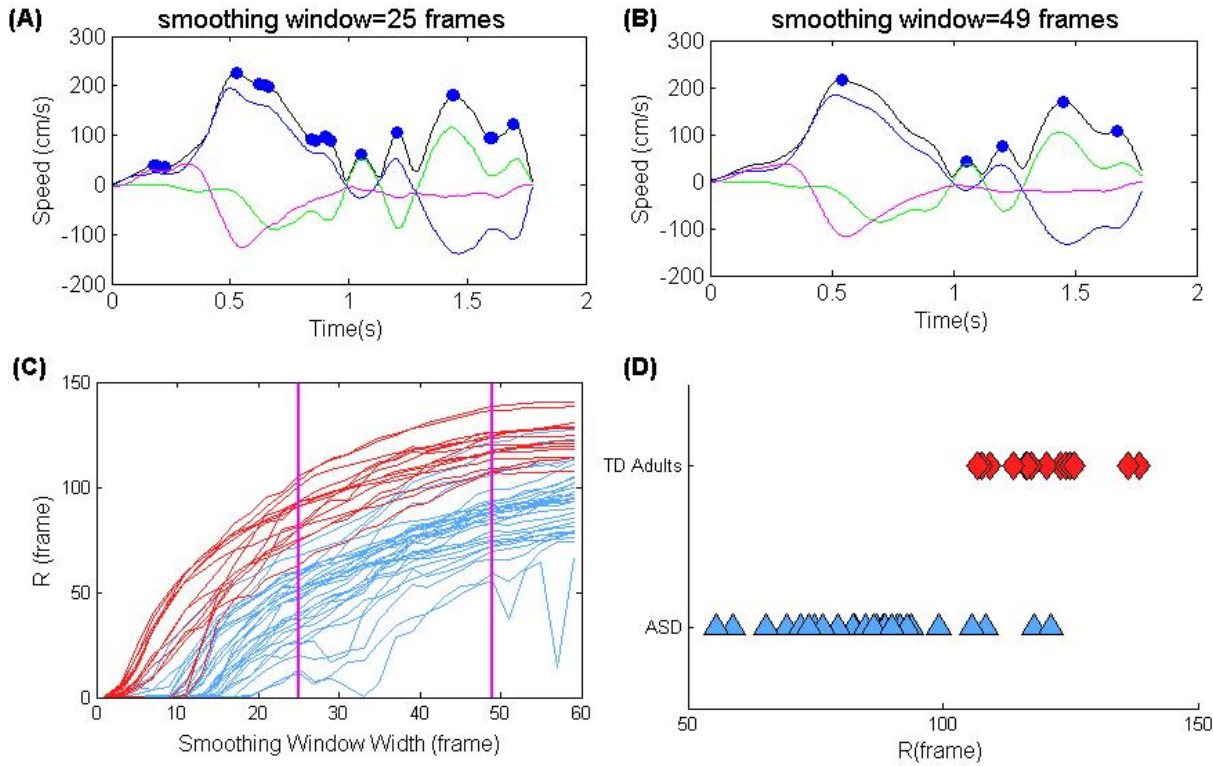

Fig. S3. Smoothing parameter selection shows robustness of the clustering results (A-B) Speed profile examples, with their corresponding filtered velocities for window widths (A) 25 frames; (B) 49 frames. The blue dots mark the corresponding local maxima in the speed profiles for each case. Some speed maxima (s-Peaks) in case (A) are missing in (B) when the smoothing window width is doubled. (C) Plot the R values for all subjects as a function of the smoothing window width. The distinction between groups is valid for a wide region of window width parameter selection, validating strongly the robustness of our results. The smoothing window width used in this paper was chosen as 25 frames (104 ms). This parameter was selected to have the two groups well separated before the R-values saturate. The results obtained when doubling the smoothing window width (to 49 frames) are provided in the plot (D). (D) Same parameter plot as Fig. 5A in the main body of the paper for results when we double the smoothing width to 49 frames. Note that the R-parameter values between ASD and TD adults are still clearly separable: the p value (t-test) is below 10^{-6} .

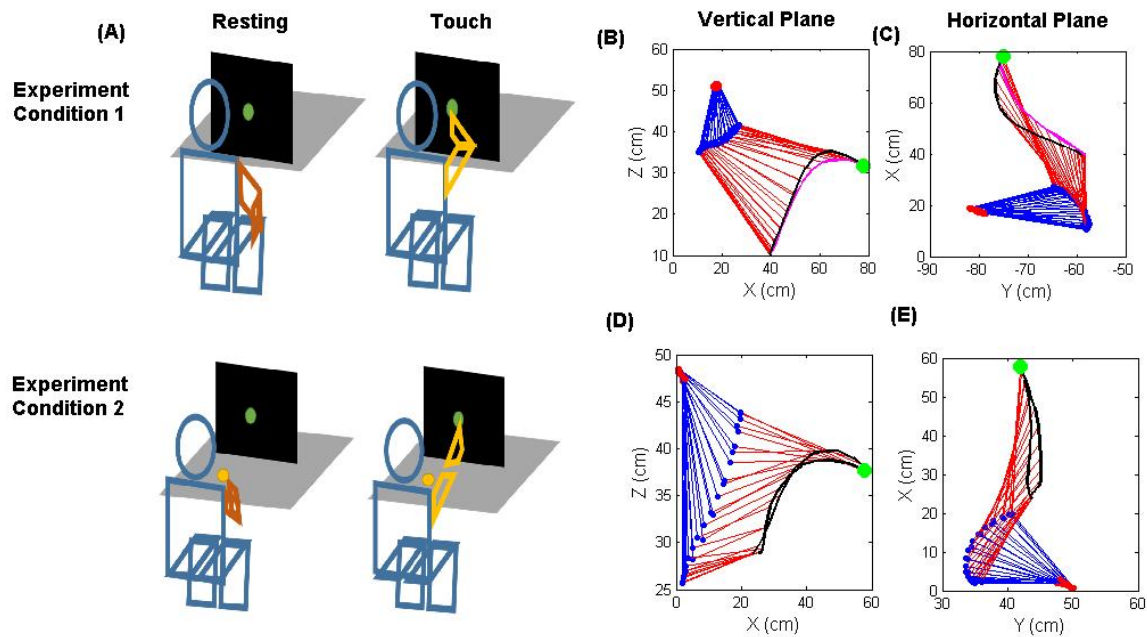

Fig. S4. Two experimental conditions (A) the cartoons illustrate the two experimental conditions. The subjects sat in front of the screen (black). In Experimental condition-1 (first row), the screen was placed at the edge of the table (grey). In experiment condition-2(second row), the screen was moved further to the middle of the table. Under condition-1, the subjects were totally free to move their arms and hands back and forward. They normally rested their hands on their lap or the chair arm (upper left panel). When they reached the target, they did not necessarily have to all stretch their arms (upper right panel). The full motion was not restricted. Under condition-2, the subjects were restricted to move their arm above the table. They had to retract their hands back to a fix resting point (yellow point) (bottom left panel). When they reached the target (bottom right panel), they often had to stretch their arm at almost full length. (B-E) plots the arm avatar for one reaching and retracting cycle for each condition. The red dot is the position of the shoulder. The blue dots in between blue and red lines are the positions of the sensors placed in the upper arm (just above the elbow) providing an estimation of elbow positions. The blue lines and the red lines estimate the position and direction of the upper arms and lower arms, accordingly. The green dot indicates the target and the black line the hand motion trajectory. (B) and (D) are the trajectories viewed from the right side (in the vertical plane). A comparison between the two conditions shows that the elbow angle changed in a wider range of values and the arm is more stretched under condition-2. (C) and (E) are the trajectories viewed from above (in the horizontal plane). Under condition -1, the elbow is to the right of the shoulder (away from the body) during the motion. In contrast, under condition-2, the elbow is in the opposite direction. The arm configurations are different under the two different conditions. However, the R values are not, as shown in Fig. 7(C) in the main paper.

Supplementary Tables

Table S1. Scores from clinical assessments and R-parameter results of ASD participants

xepui	Gender	Age	Q		ADI_R				ADOS						Vineland						R<br>(Frames,<br>1/240s)
					A. Qual. Impairments in Reciprocal Social Interaction	B. Qual. Abnormalities in Communication	C. Restricted, Repetitive Stereotyped Patterns of Behaviors	D. Abnormality of development at or before 36 months	Module	Sect. A: Language and Communication	Sect. B: Reciprocal Social Interaction	Sect. C: Play	Sect. D: Stereotyped Behavior And Restricted Interests	Communication and Social Interaction Total	Communication Domain	Daily Living Skills Domain	Socialization Domain	Motor Skill Domain	Adaptive Behavior Composite		
1	F	22	NA	NA	-	-	-	2	1	6	14	4	2	20	25	45	27	-	19	24.9	
2	M	12	95	3-Leiter-R	23	16 (V)	7	5	1	5	8	2	2	13	55	49	50	87	58	25.9	
3	M	30	36	3-Leiter-R	29	14(V)	10	5	2	8	14	2	2	22	19	19	19	-	19	33.2	
4	M	15	56	3-Leiter-R	28	14(NV)	4	4	1	4	10	2	3	14	40	26	52	-	36	33.4	
5	F	15	71	3-Leiter-R	20	13(V)	8	4	2	5	7	0	6	12	39	19	52	-	34	33.5	
6	F	15	52	3-Leiter-R	22	22(V)	5	2	3	6	11	1	2	17	84	46	73	29	-	35.9	
7	M	13	89	3-Leiter-R	26	24(V)	5	5	2	3	7	1	2	10	50	32	50	-	41	39.9	
8	M	18	76	3-Leiter-R	30	18(V)	7	5	2	5	7	2	1	12	56	19	45	-	37	41.8	
9	M	18	101	1-Wisc-III	28	19(V)	4	5	3	4	6	1	2	10	52	48	50	-	46	46.5	
10	M	12	67	3-Leiter-R	21	14(NV)	10	3	1	5	13	4	4	18	75	72	74	69	67	47.3	
11	M	10	107	3-Leiter-R	12	7(NV)	5	5	1	3	9	4	3	12	57	61	72	97	66	48.4	
12	F	16	81	1-Wisc-III	23	18(V)	6	5	3	7	9	1	2	16	58	50	66	-	53	50.8	
13	F	12	NA	NA	21	14(NV)	9	5	1	8	10	4	4	18	55	60	53	73	55	51.9	
14	F	15	77	3-Leiter-R	27	21(V)	8	4	2	7	11	8	4	18	79	69	42	56	39	52.2	
15	M	18	96	1-Wisc-III	23	13(V)	6	5	3	4	8	0	4	12	66	60	53	-	55	55.4	
16	M	14	74	3-Leiter-R	20	10(NV)	10	5	1	9	10	4	3	19	47	38	53	76	49	57.7	
17	M	25	99	5-wais-III	24	8(V)	8	3	4	3	7	1	3	10	109	121	85	-	107	58.0	
18	M	12	95	3-Leiter-R	11	13(V)	7	3	2	5	7	1	1	12	73	58	76	102	71	71.4	

The 1-WISC-III(Weschler Intelligence Scale for Children-III) ², the 5-WAIS-III (Weschler Adult Intelligence Scale-III)³ and the 3-Leiter-R (Leiter International Performance Scale)⁴ were used to assess the intelligence of the participants with ASD. IQ score is a standardized score (M = 100, SD = 15) for intelligence level. The ADI-R (Autism Diagnostic Interview-Revised) ⁵ and the ADOS (Autism Diagnostic Observational Scale) ^{6,7} are standard assessment tools used by clinicians as a basis for the ASD diagnosis. Vineland Adaptive Behavior scale⁸ is a standardized measure of adaptive behavior (M = 100, SD = 15).

Table S2. ASD participants with high/low functioning labels

Index	Gender	Age (yrs)	category	R (frames)
1	M	13.8	High	68.3
2	M	11.5	High	58.9
3	M	11.7	High	60.2
4	F	14.3	High	51.1
5	M	7.6	High	52.7
6	M	9.9	High	61.1
7	M	11.7	Low	11.7
8	F	7.8	Low	37.1
9	F	>14	Low	13.7
10	F	>14	Low	27.5
11	M	>14	Low	20.2

Subjects 9-11 are teenagers with ages older than 14. Their precise ages were missing from the records.

Table S3. Parent's Information

ASD Subject Index		Mother Age		Father Age
2	Mother	35	Father	35
3	Mother	58	Father	58
5	Mother	44		
7	Mother*	36		
8	Mother	55	Father	52
9	Mother	49		
10	Mother	40	Father*	54
11	Mother	38	Father	38
12	Mother	52		
13	Mother	44	Father	50
15	Mother*	37		
17	Mother	58		
18	Mother	38	Father*	39

*Father of #10 subject was a late talker. Father of #18 subject reported eye and motor coordination problems, ADHD and delayed reading. Mother of #15 subject had dyslexic problem. Mother of #7 subject reported slower reading problems. Father of #7 subject (data not collected) had record of bipolar and borderline personality. The other parents had no noted problems. The three parents falling into the TD region in the R-space were gray shaded. The other parents all fell into the ASD region.

Table S4. Information from TD participants

Participant	Gender	Age
1	M	3
2	M	4.3
3	F	4.3
4	F	4.8
5	M	4.8
6	M	5.1
7	F	19
8	F	20
9	F	21
10	F	21
11	M	22
12	M	22
13	M	24
14	F	24
15	F	24
16	M	24
17	F	25
18	F	27
19	M	27
20	M	31
21	M	31

Typically developing children (1-6) and typical controls (shaded in gray).

Caption of Supplementary Movie

SI movie 1

Animations of hand movement for (A) Case 1 ASD (B) Case 3 ASD and (C) a typical developing adult and their corresponding speed profiles (D-F) in the pointing task. The animation was sampled at 24Hz and played at half of the actual speed (12Hz). Time stamp of each frame was indicated at the top right corner. In (A-C), the black triangles represent the pointing hands' positions and the green dots represents the targets. There is no significant visual differences among the subjects when they are completing reaching forward and retracting backward cycles. Smoothness differences appear in their speed profiles in (D-F). Only the motion profiles inside motion cycles (colored as blue, purple and red, same code as the figures in the text) are included in the analyses of this paper.

References

- 1 Butterworth, S. On the theory of filter amplifiers. *Wireless Engineer* **7**, 536-541 (1930).
- 2 Wechsler, D. *WISC-III: Wechsler intelligence scale for children: Manual*. (Psychological Corporation, 1991).
- 3 Wechsler, D. *WAIS-III: Wechsler adult intelligence scale*. (Psychological Corporation, 1997).
- 4 Roid, G. H. & Miller, L. J. *Leiter-R: Leiter International Performance Scale*. (Stoelting, 2002).
- 5 Rutter, M., Le Couteur, A. & Lord, C. Autism diagnostic interview-revised. *Los Angeles, CA: Western Psychological Services* **29**, 30 (2003).
- 6 Lord, C. *et al.* The autism diagnostic observation schedule-generic: a standard measure of social and communication deficits associated with the spectrum of autism. *J Autism Dev Disord* **30**, 205-223 (2000).
- 7 Gotham, K., Pickles, A. & Lord, C. Standardizing ADOS scores for a measure of severity in autism spectrum disorders. *J Autism Dev Disord* **39**, 693-705, doi:10.1007/s10803-008-0674-3 (2009).
- 8 Sparrow, S. S., Balla, D. A., Cicchetti, D. V., Harrison, P. L. & Doll, E. A. Vineland adaptive behavior scales. (1984).
